# New Method of Operating

**Published:** 1848-01

**Authors:** 


### NEW METHOD OF OPERATING.

Take a common low rocking-chair, on which place your patient. Let him open wide his mouth. The operator then with one hand holds in his mouth the mirror, and with the other handles his instruments. This is applicable to only the upper teeth. We are told that it avoids the necessity of stooping, the glass reflecting the teeth so that the operator can see what he is at without stooping to get a direct view of the cavity, as in ordinary cases. The above plan is by one of our young Dentists, and when *patented* will be offered for use to the profession.—Cin. Ed.

